# A threshold model for receptor tyrosine kinase signaling specificity and cell fate determination

**DOI:** 10.12688/f1000research.14143.1

**Published:** 2018-06-21

**Authors:** Allen Zinkle, Moosa Mohammadi

**Affiliations:** 1 Department of Biochemistry & Molecular Pharmacology, New York University School of Medicine, New York, NY, USA

**Keywords:** Receptor tyrosine kinase, ligand-induced receptor dimerization, signaling intensity and duration

## Abstract

Upon ligand engagement, the single-pass transmembrane receptor tyrosine kinases (RTKs) dimerize to transmit qualitatively and quantitatively different intracellular signals that alter the transcriptional landscape and thereby determine the cellular response. The molecular mechanisms underlying these fundamental events are not well understood. Considering recent insights into the structural biology of fibroblast growth factor signaling, we propose a threshold model for RTK signaling specificity in which quantitative differences in the strength/longevity of ligand-induced receptor dimers on the cell surface lead to quantitative differences in the phosphorylation of activation loop (A-loop) tyrosines as well as qualitative differences in the phosphorylation of tyrosines mediating substrate recruitment. In this model, quantitative differences on A-loop tyrosine phosphorylation result in gradations in kinase activation, leading to the generation of intracellular signals of varying amplitude/duration. In contrast, qualitative differences in the pattern of tyrosine phosphorylation on the receptor result in the recruitment/activation of distinct substrates/intracellular pathways. Commensurate with both the dynamics of the intracellular signal and the types of intracellular pathways activated, unique transcriptional signatures are established. Our model provides a framework for engineering clinically useful ligands that can tune receptor dimerization stability so as to bias the cellular transcriptome to achieve a desired cellular output.

## Introduction

The receptor tyrosine kinase (RTK) superfamily comprises 58 single-pass transmembrane proteins. These receptors signal in response to extracellular stimuli delivered by over 100 different ligands (growth factors, cytokines, and hormones) and thereby govern a myriad of essential biological processes throughout an organism’s life span. RTK signaling is required for every event during embryonic development, including gastrulation, mesoderm induction, organogenesis, tissue patterning, and body plan formation ^1^. It also regulates energy and mineral metabolism ^2^, immune responses ^3^, tissue homeostasis ^4^, and a wide spectrum of other functions. The diversity of RTK actions is reflected by the fact that both gain- and loss-of-function mutations in RTKs are causative of a diverse array of developmental, metabolic, and autoimmune diseases ^5^ as well as cancer ^6^.

The insulin receptor (IR), vascular endothelial growth factor receptor, fibroblast growth factor receptor (FGFR), epidermal growth factor receptor (EGFR), nerve growth factor receptor (NGFR), stem cell factor (SCF) receptor c-Kit, and other RTKs each possess an extracellular ligand-binding domain, a single transmembrane helix, and an intracellular kinase domain which is flanked at either end by juxtamembrane (JM) and C-terminal tail regions. Based on differences in the overall architecture of their extracellular domains, RTKs are grouped into 20 subfamilies ^7^. The extracellular domain of each subfamily features a unique configuration of domains such as Ig-like, fibronectin-like, leucine-rich, cysteine-rich, and other modular domains that are specialized for the recognition of distinct ligands such as EGF, platelet-derived growth factor (PDGF), insulin, NGF, and FGF. In contrast, the intracellular kinase domains, which catalyze the transfer of γ-phosphate from ATP to substrate tyrosine residues in both the receptor itself and in intracellular downstream substrates, are structurally homologous. With the exception of members of the IR subfamily, which are preformed dimers ^8^, all RTKs are monomeric in the absence of ligand and rely on a process of ligand-induced dimerization to elevate the intrinsic activity of the intracellular kinase domain. In these cases, ligand-induced dimerization of the extracellular domain of RTKs juxtaposes the intracellular kinase domains in a precise orientation conducive to transphosphorylation of one or more tyrosines in the kinase activation loop (A-loop). This transphosphorylation activates the kinase in an allosteric fashion ^9^. In the case of the IR subfamily, ligand binding reorients the subdomains within the extracellular domain of the preformed receptor dimer. This structural rearrangement is propagated through the transmembrane helices and enables transphosphorylation of A-loop tyrosines ^10^. Some RTKs, including PDGF receptor beta (PDGFRβ), c-Kit, and fms-like tyrosine kinase 3 (FLT3), are activated by transphosphorylation of JM tyrosines, although they still depend on A-loop tyrosine phosphorylation to exert their full biological activities ^11– 13^. In the case of the EGFR subfamily, A-loop phosphorylation also plays a secondary role in receptor activation; EGFRs are activated via the formation of an asymmetric kinase dimer, whereby an activator kinase allosterically stabilizes the active conformation of a receiver kinase ^14^.

**Figure 1.**
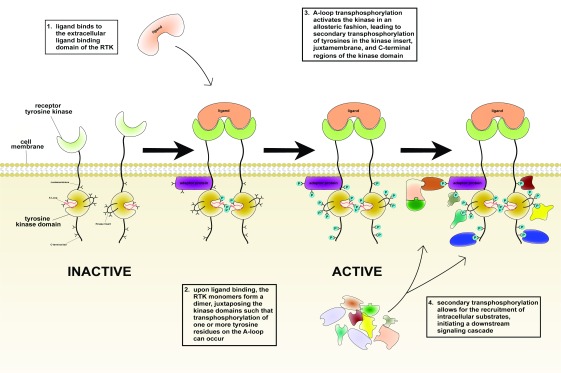
Upon ligand binding, receptor tyrosine kinases (RTKs) dimerize and become activated. From left to right: ligand binding induces RTK dimerization, thus juxtaposing the intracellular kinase domains in a proper orientation/proximity such that transphosphorylation of tyrosines (shown as Y symbols) in the activation loop (A-loop), and hence kinase activation, can occur. This in turn triggers secondary transphosphorylations (shown in turquoise) in the kinase insert, juxtamembrane, and C-terminal tail regions, creating docking sites for the recruitment of distinct intracellular substrates (shown in an assortment of shapes/colors on the lower right). Darker shades are used to denote bound and activated substrates in the right-hand cartoon.

A-loop-dependent or -independent kinase activation triggers transphosphorylation of tyrosine residues located in the kinase insert region as well as in the JM and C-terminal tail regions ^15^. These secondary phosphorylations play a critical role in signal transduction by creating specific docking sites for Src homology domain 2 (SH2)- or phosphotyrosine-binding domain (PTB)-containing cytosolic or membrane-anchored substrates (enzymes, adaptors, and scaffold proteins) ^16^, thereby physically recruiting the substrate to the activated RTK. In the case of substrates endowed with enzymatic activity, such as phospholipase Cγ (PLCγ), this recruitment plays a dual role: 1) it facilitates phosphorylation and hence upregulation of the intrinsic activity of the enzyme, and 2) it brings the activated enzyme into proximity with its substrate—in this case, phosphatidylinositol 4,5-bisphosphate (PIP2)—in the plasma membrane ^17^. By contrast, recruitment of adaptors or scaffold proteins that lack enzymatic activity, such as FGFR substrate 2 alpha (FRS2α), solely facilitates their phosphorylation. Phosphorylated adaptor proteins serve as molecular hubs for the coordinated assembly of signaling complexes at the cell membrane close to their substrates ^18^. A prime example of such events is the recruitment of the growth factor receptor-bound protein 2-son of sevenless (Grb2-Sos) complex next to membrane-associated Ras ^19, 20^. In other cases ^21^, these indirect recruitments facilitate phosphorylation and activation of substrates such as SH2-containing protein tyrosine phosphatase 2 (Shp2) by the RTK itself. Such recruitment/phosphorylation events trigger activation of multiple downstream pathways, altering the transcriptional profile of the cell to influence cellular proliferation, differentiation, migration, apoptosis, metabolism, and senescence ( Figure 1).

Despite exerting a diverse array of biological responses, there are only a handful of intracellular pathways known to operate downstream of activated RTKs. Examples include the Ras/mitogen-activated protein kinase (Ras/MAPK) ^22^, phosphoinositide 3-kinase/protein kinase B (PI3K/PKB) ^17^, c-Jun N-terminal kinase (JNK) ^23^, P38 MAPK, Rac, PLCγ/protein kinase C (PKC) ^17^, and Janus kinase/signal transducer and activator of transcription (JAK/STAT) pathways ^24^. Activation of these pathways leads to changes in the cellular expression/phosphorylation status of numerous transcription factors ^15^, ultimately culminating in distinct cellular responses/fates. Relative to intracellular pathways, transcription factors are more numerous, suggesting that each signaling pathway theoretically could act through a unique subset of these factors. However, different pathways often converge on common sets of transcription factors. For instance, the Ras/MAPK and PI3K/Akt signaling pathways share cAMP response element-binding protein (CREB), estrogen receptors, and GATA2 as transcription factors ^25– 28^. Accordingly, how RTKs elicit their distinct cellular/developmental responses through the use of a shared set of intracellular pathways constitutes a puzzle that has preoccupied researchers in the field for at least two decades ^29^.

An initial solution to this conundrum was provided in a now-classic paper by C.J. Marshall, who proposed that the overall duration and intensity of RTK-induced intracellular signaling pathways (more specifically, MAPK activation), and not a specific pathway per se, are the primary determinants of the cellular response ^22^. However, later studies found that, depending on the nature of their cognate ligand, RTKs undergo transphosphorylation on distinct tyrosines, resulting in recruitment/activation of ligand-specific intracellular pathways and cellular responses ^30, 31^, thus supporting the concept of one pathway per cellular activity. This latter hypothesis has the advantage of simplicity but is undermined by the fact that there exist more RTK-mediated cellular outputs than intracellular pathways. Therefore, a holistic model for RTK signaling must consolidate the seemingly discordant ideas that RTKs use signaling intensity/duration as well as distinct intracellular pathways to determine specificity or diversity or both. Recent studies have suggested that the primary determinant of the cellular response may be the strength of receptor dimerization (dimer stability) ^32, 33^, defined by the thermodynamics of dimerization (that is, on- and off-rates). This in turn is governed by RTK-specific multivalent protein–protein binding events involved in dimer assembly, including (but not limited to) ligand–receptor, receptor–receptor, co-receptor–ligand, and co-receptor–receptor interactions. The aims of this review are to revisit previously existing models for RTK signaling and to synthesize a more comprehensive model that integrates past results with more recent findings. We also briefly present future directions for studying RTK signaling.

## Role of signaling intensity and duration in receptor tyrosine kinase-mediated cellular and developmental processes

C.J. Marshall used the rat adrenal pheochromocytoma (PC12) cell line—which naturally expresses NGFR and EGFR—as a model system to show that RTKs can determine cellular fates by regulating the dynamics (that is, amplitude and duration) of MAPK pathway activation ^22^. Treatment of PC12 cells with NGF causes sustained MAPK activation, giving rise to neurite outgrowth, whereas treatment of the same cells with EGF leads to transient MAPK phosphorylation, inducing cellular proliferation. However, forced overexpression of EGFR enables EGF to produce persistent MAPK activation and induce neurite outgrowth. Conversely, reducing the number of NGFR molecules per cell leads to transient MAPK activation and proliferation by NGF. These data led Marshall to conclude that the duration and amplitude of MAPK activation, and not RTK or ligand identity, are the primary determinants of cellular responses.

Marshall’s hypothesis has since gained momentum by numerous studies, particularly on the FGF system. This is perhaps best exemplified by the functional dichotomy between FGF8a and FGF8b, two alternatively spliced FGF8 isoforms, which use the Ras/MAPK pathway as the nexus to pattern the midbrain ^34^. The “b” isoform of FGF8 (FGF8b) induces differentiation of the midbrain into cerebellum, whereas the “a” isoform (FGF8a) lacks this effect, instead causing expansion (proliferation) of the midbrain. However, FGF8b can be functionally converted to FGF8a by simply reducing its expression level ^35^. Since changing FGF ligand concentration naturally affects the extent of receptor phosphorylation and accompanying downstream intracellular signaling, these data imply that signaling strength, rather than ligand identity, determines the nature of neuronal patterning. Indeed, follow-up quantitative analysis in the same study revealed that FGF8b produces a MAPK signal two orders of magnitude stronger than that of FGF8a ^35^. Thus, reminiscent of PC12 cell differentiation, differences in the neuronal patterning capacities of FGF8a and FGF8b can be traced to corresponding differences in the magnitude of the Ras/MAPK signal transmitted by these two isoforms. Consequently, a strong Ras/MAPK signal elicited by FGF8b is necessary to cause differentiation of midbrain into cerebellum, whereas a weak signal sent by FGF8a leads to expansion of the midbrain ^36^. Similarly, a study of inner ear development in mouse embryos revealed the existence of a quantitative threshold for FGF signaling necessary for otic vesicle formation ^37^. Moreover, a gene knockout study in mice designed to dissect the role of four FGF family members expressed by the apical ectodermal ridge (AER) found that inactivation of *FGF4*, *9*, and *17* either individually or in combination with *FGF8* results in skeletal phenotypes of increasing severity ^38^. These data imply that each FGF contributes to the total FGF signal emanating from the AER, whose magnitude must meet a certain threshold in order to induce/maintain proper limb development. Similarly, successive knockdown of the E26 transformation-specific (ETS) family transcription factors *Erm*, *Etv5*, and *Pea3* in the zebrafish embryo results in an increase in cardiac progenitors, and the blocking of all three genes in turn results in the suppression of FGF target genes ^39^. Because *Pea3* contains a MAPK-specific phosphorylation site also present in both *Erm* and *Etv5* ^40^, this indicates that all three factors are regulated by MAPK activation. Hence, FGF-induced MAPK signaling dynamics may determine the landscape of activated ETS factors, in turn establishing distinct events in zebrafish development.

A similar conclusion can be drawn from gene knock-in studies in mice designed to dissect the roles of intracellular pathways in mediating particular cellular actions of other RTKs ^41– 43^. One of these studies ^43^ examined the roles of five different pathways downstream of PDGFR in the development and maintenance of vascular smooth muscle cells/pericytes; the approach involved the generation of a PDGFRβ allelic series in mice carrying progressive mutations of tyrosines that mediate the recruitment/activation of these intracellular pathways. The severity of the loss of vascular smooth muscle cell/pericyte population was found to correlate with the number of intracellular pathways inactivated. Manipulation of PDGFR expression levels also showed a quantitative relationship between receptor expression levels and the vascular smooth muscle phenotype. Additional support for Marshall’s model has been provided by cell-based studies showing that changing the concentration (and thus the signaling strength) of FGF2 or FGF8, and FGF9, respectively, induces distinct responses in pre-somitic mesoderm and primordial germ cells ^44, 45^.

A hallmark of the Marshall model is that neither receptor nor ligand identities are relevant to the cellular response: rather, the signaling intensity, duration, and, more specifically, the extent of MAPK activation are all that matters. Indeed, it has been shown that many RTKs are interchangeable in exerting a particular biological function. This is exemplified by data showing that tracheal migration defects seen in DFGF-R mutant fly embryos can be partially rescued by a constitutively dimeric allele of Torso, a *Drosophila* RTK homologous to human PDGFR. Moreover, swapping the intracellular kinase domain of this constitutively dimeric allele of Torso with those of other fly RTKs, including DFGF-R1, DFGF-R2, DER (the *Drosophila* homolog of EGFR), and Sevenless (an RTK most similar to IR and IGF1R), does not affect the rescue capacity of dimeric Torso ^46^. The ability of heterologous RTKs to mimic the action of DFGF-R in tracheal development argues strongly in favor of the involvement of an overlapping set of intracellular pathways in tracheal development.

Consistent with data garnered from multicellular systems, a recent study in yeast has shown that exposure to different dosages of mating pheromone activates MAPK to different extents so as to elicit distinct cellular responses ^47^. Specifically, a high pheromone dose induces sustained activation of Fus3—a downstream MAPK protein—resulting in growth arrest and the formation of a pear-shaped morphology. Conversely, a low pheromone dosage leads to transient Fus3 activation and elongated cell growth. Hence, it appears that the regulation of cellular fates by ligand-induced changes in MAPK dynamics is an evolutionarily conserved mechanism.

## Distinct pathways mediate specific cellular functions

Although mounting evidence exists in support of the Marshall model, a wealth of cell-based evidence suggests that RTKs use distinct intracellular pathways to exert their specific cellular activities. Importantly, many studies have dissected the role of intracellular pathways in a given biological readout by ablating the docking sites on RTKs for distinct SH2/PTB-containing intracellular substrates. For example, elimination of a conserved tyrosine residue in the C-terminal tail of FGFR prevents the receptor’s ability to recruit, phosphorylate, and activate the PLCγ/PKC pathway. This mutation has no impact on FGF-induced MAPK activation, mitogenesis of L6 myoblasts ^48^, or the differentiation of PC12 cells ^49^, but it does impair FGFR internalization ^50^. Hence, the PLCγ/PKC pathway is evidently dispensable for mitogenic/differentiation responses to FGFs, but it is essential for receptor endocytosis/trafficking. In contrast, it has been shown that FGF-induced mitogenesis and differentiation depend on the efficient recruitment/phosphorylation of FRS2α, an adaptor protein that links FGFR activation to the MAPK and PI3K pathways. Specifically, disruption of the docking site for the PTB domain of FRS2α in the JM region of FGFR1 impairs FRS2α binding to FGFR1, leading to reduced tyrosine phosphorylation of FRS2α and about a 30–40% reduction in MAPK activation ^51^. Moreover, FGF treatment of FRS2α-deficient fibroblasts completely failed to induce MAPK activation, cellular proliferation, and migration ^52^. Notably, in the same study, rescue experiments using wild-type and mutated FRS2α constructs carrying mutations in the docking sites for Grb2 and Shp2 showed that the latter two proteins synergistically contribute to FGF-induced MAPK activation, cellular proliferation, and migration. These impairments are due to the inability of the mutant FRS2α constructs to translocate the Grb2-Sos complex (a Ras GTP exchange factor) into the vicinity of its substrate (Ras) in the plasma membrane ^53^. Intriguingly, the additive effects of Grb2 and Shp2 binding to FRS2α in activating MAPK pathways and promoting cellular proliferation and migration are reminiscent of the additive effects of downstream pathways in PDGFR-mediated regulation of vascular smooth muscle cells/pericytes in mice ^43^. Together, these data show that FGF-induced mitogenesis and differentiation are inextricably linked to FRS2α-mediated activation of the Ras/MAPK pathway. Ablation of the FRS2α docking site on FGFR had no effect on activation of the PLCγ/PKC pathway ^52^, confirming earlier data showing that PLCγ/PKC pathway activation is dispensable for FGF-induced proliferation and differentiation.

In the case of PDGFR, mutations of five tyrosine residues located in the kinase insert and C-terminal tail regions of the receptor—which together are responsible for the recruitment and activation of PLCγ, the Ras GTPase-activating protein (RasGAP), and the P85 subunit of PI3K—have been shown to compromise the ability of PDGFR to conduct mitogenesis ^54^. Upon selectively reinstating the tyrosine residues that mediate PLCγ and PI3K recruitment/activation, respectively, mitogenic functionality could be fully restored. In contrast, reinstating the tyrosine residue that mediates RasGAP recruitment/activation alone failed to rescue mitogenesis.

In another example, ablation of the Grb2-recruitment site and application of wortmannin (a PI3K-selective inhibitor ^55^) were each used to dissect the roles of the Ras/MAPK and the PI3K pathways in mediating cell dissociation/scattering and branching tubulogenesis by MET (the receptor for hepatocyte growth factor/scatter factor [HGF/SF]) in MDCK (Madin-Darby canine kidney) epithelial cells ^56^. Although the inhibition of PI3K eliminated the ability of HGF to induce cell dissociation/scattering, it had no impact on HGF-induced tubulogenesis. Conversely, disruption of Grb2 recruitment (inhibition of MAPK) eliminated HGF-induced tubulogenesis without affecting the ability of HGF to induce scattering of MDCK cells. Hence, MET-mediated cell dissociation/scattering and branching tubulogenesis appear to be pathway dependent and not contingent on signaling dynamics.

The hypothesis that RTKs rely on non-overlapping pathways to exert their cellular actions is reinforced by data showing that ligands that signal through a shared receptor selectively recruit/activate particular intracellular substrates to elicit their distinct functions. For example, immunofluorescence and immunoelectron microscopy analyses in epithelial cells have shown that whereas FGF7 stimulation of FGFR2b causes receptor degradation and cell proliferation, FGF10 stimulation of the same receptor leads to receptor recycling and cell migration ^57^. Mechanistically, it appears that FGF10 binding to FGFR2b induces receptor phosphorylation of a particular tyrosine residue, Y734, which the binding of FGF7 cannot achieve. When phosphorylated, this residue mediates recruitment of a complex consisting of P85-PI3K and the adaptor protein SH3 domain-binding protein 4 (SH3BP4) to FGFR2b. Mutation of Y734 to phenylalanine switches the FGF10 response into an FGF7-like response, thereby implicating this complex as the molecular conduit for FGF10-mediated effects ^58^.

Other studies that did not depend on the mutation of recruitment sites for intracellular substrates have also shown qualitative associations between intracellular pathways and cellular responses ^59^. For example, while FGFR4 employs the PLCγ pathway to induce cardiac hypertrophy in chronic kidney disease, it also activates the JNK pathway to regulate bile acid synthesis in hepatocytes ^60, 61^. Similarly, as a matter of preference between RTK and choice of pathway, PDGF and FGF each use distinct pathways in order to activate MAPK to comparable levels in mouse fibroblasts. PDGFR accomplishes this by exhibiting a strong preference for PI3K-dependent pathways, whereas FGFR, which is only a very weak activator of PI3K, activates MAPK through relatively more potent Ras-dependent pathways ^62^. Furthermore, comparison of the transcriptional response of mouse embryonic palatal mesenchyme cells with PDGF and FGF revealed that the PDGF response is PI3K dependent and promotes differentiation but that the FGF response is MAPK dependent and favors proliferation ^31^. Even within the same RTK subfamily, differences in preference for downstream pathways are persistent. For example, FGFR1 favors the Ras/MAPK pathway, whereas FGFR4 signals mostly through PLCγ ^63^. Thus, although some RTKs seem to require specific pathways for carrying out a distinct function, like PDGFR with respect to PLCγ/PKC and mitogenesis, other receptors such as FGFR1 find these pathways dispensable for the same function.

## A unifying model for regulation of receptor tyrosine kinase signaling specificity

**Figure 2.**
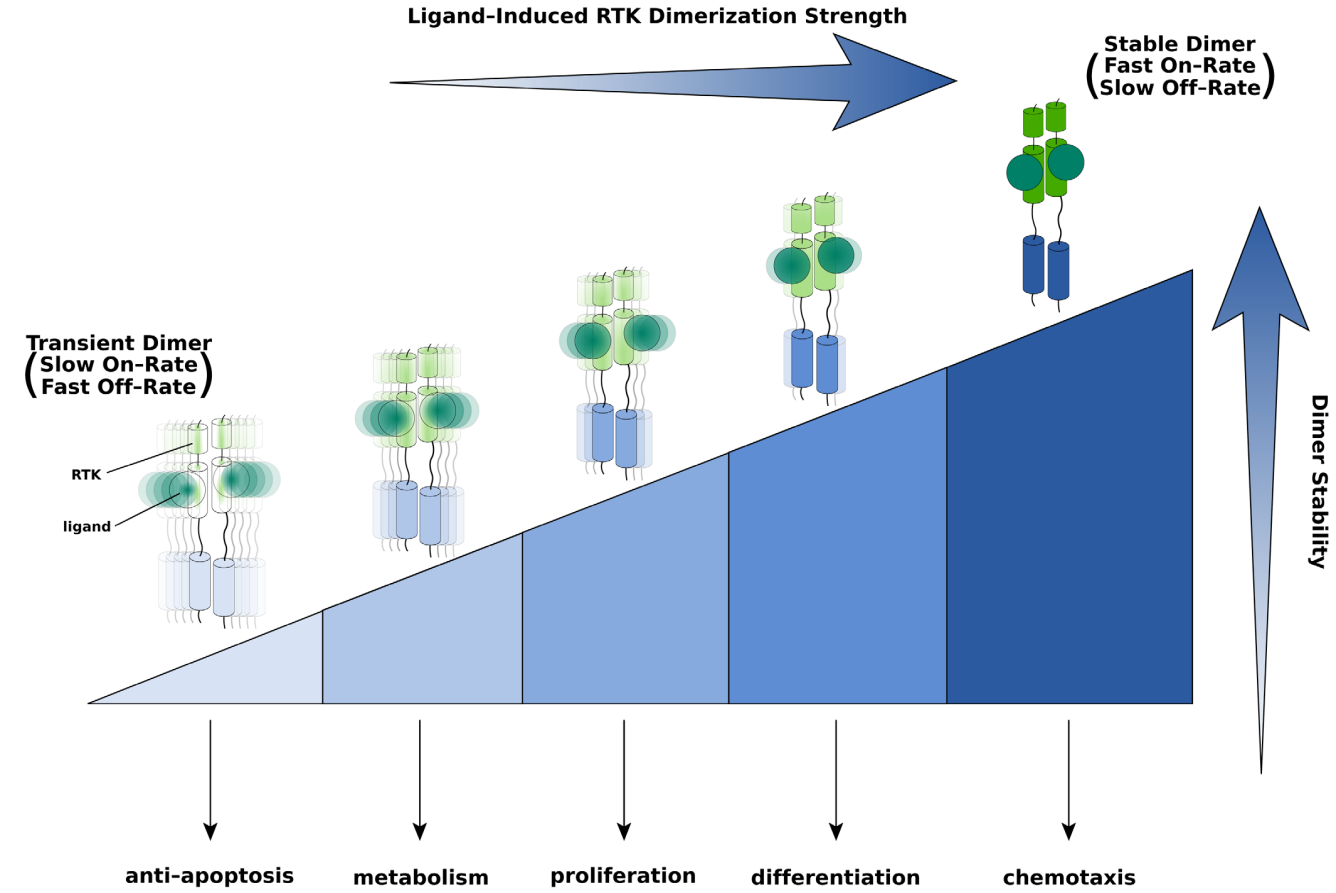
A unifying threshold model for receptor tyrosine kinase (RTK) signaling specificity and cell fate determination. Different thresholds in dimer strength/stability are required to alter the transcriptional landscape and cellular fate. The strongest/most-stable dimer—at the right-hand end of the threshold spectrum (depicted by the shaded/sectioned triangle)—is able to perform any and all cell responses to its left. By contrast, the weakest/less-stable dimer—pictured at the left end of the spectrum—is capable only of eliciting a single response. The on- and off-rates of a prototypical ligand-induced RTK dimer (that is, dimer stability) are determined by the energetics of ligand–receptor, receptor–receptor, and other protein–protein binding events specific to a given RTK. Based on the model, therapeutically useful ligands can be engineered by fine-tuning receptor dimer stability through changes in the strengths of individual protein–protein interactions involved in dimerization. Intensity of color and sharpness of focus as well as oscillations of the dimer are used to emphasize differences in dimer strength/stability (that is, on- and off-rates) and rigidity.

The data summarized above clearly support the view that RTKs can dictate cellular responses by transducing intracellular signals that are both qualitatively and quantitatively distinct. Our cumulative insights into the structural biology of FGF signaling have enabled us to formulate a “threshold” model for RTK signaling specificity that unifies the wealth of literature highlighting the existence of a link between specific cell fates and quantitative and qualitative differences in intracellular signals. Crystal structures of FGFR kinase transphosphorylation complexes and associated steady-state kinase assay data have revealed kinetic differences in the “phosphorylability” of different tyrosines ^64– 67^; that is, some sites appear kinetically disadvantaged over others. This implies that different phosphorylation sites require different degrees of dimer stability in order to become phosphorylated. It follows that the extent and pattern of tyrosine phosphorylation in a given RTK are directly governed by different thresholds in durability/strength of the RTK dimer. More specifically, compared with a strong/stable dimer, a weak/transient RTK dimer is unable to phosphorylate kinetically disadvantaged sites and also phosphorylates fewer A-loop and recruitment-site tyrosines overall. In contrast, a strong/persistent RTK dimer robustly phosphorylates/activates both A-loop and kinetically disadvantaged recruitment-site tyrosines, thereby quantitatively and qualitatively activating more downstream pathways. Therefore, we propose that different thresholds in dimer strength/stability enable the generation of distinct downstream signals and that these give rise to unique transcriptional landscapes that determine cellular fates. In physicochemical terms, the dimer stability—that is, the distinction between a “weak” and “strong” RTK dimer—is dictated by the on- and off-rates of dimerization. This in itself is a reflection of both the net energetic parameters of various multivalent protein–protein binding events involved in dimer assembly and the concentrations of the reactants (ligand, receptor, co-receptor, and so on). These protein–protein binding events include ligand–receptor, receptor–receptor, co-receptor–ligand, co-receptor–receptor, and other interactions that are specific to a given RTK. Intuitively, a weak/transient dimer would be expected to have a slow on-rate and a fast off-rate, whereas a strong or stable dimer would possess a relatively faster on-rate and a slower off-rate ( Figure 2).

The crystal structure of FGF8b in complex with the “c” isoform of FGFR2 (FGFR2c) has been particularly instrumental in formulating the threshold model. This structure reveals that Phe-32 from the alternatively spliced N-terminus of FGF8b, absent in FGF8a, engages in an additional hydrophobic contact with FGFR2c that confers on FGF8b an FGFR binding affinity that is an order of magnitude higher relative to FGF8a. Mutation of Phe-32 to alanine (F32A) reduces the binding affinity of FGF8b for its receptors to levels similar to that of FGF8a while functionally converting the mutant FGF8b to an FGF8a-like molecule. Specifically, in chick brain explants, the F32A FGF8b mutant failed to induce differentiation of chick midbrain into hindbrain and instead mirrored the functionality of FGF8a by causing proliferation of the midbrain ^68^. These results were confirmed at the level of midbrain gene expression: FGF8b strongly induces the expression of genes for the homeobox transcription factors En2 and Gbx2 as well as for the cytoplasmic-negative regulator Spry1. Because Gbx2 is a suppressor of Otx2, another homeobox transcription factor and a midbrain-specific marker, FGF8b also represses *Otx2* ^69^. In contrast, as in the case of FGF8a, the mutant F32A FGF8b only weakly induces *En2* and *Spry2* and completely fails to induce *Gbx2*, thus leaving *Otx2* expression unchanged.

**Figure 3.**
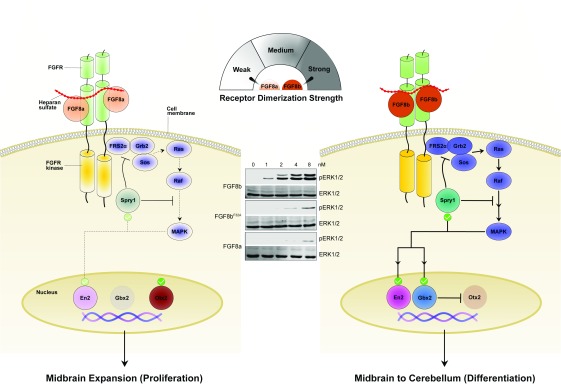
The “threshold model”, as exemplified by the FGF8-FGFR system, can explain disparities in transcriptional activity. (Left) FGF8a dimerizes its receptor weakly, thus transducing a transient signal that can only weakly induce En2 and Spry1 expression, while totally failing to induce expression of Gbx2, the Otx2 suppressor. In the absence of Gbx2, the expression of Otx2 remains high. Under these conditions, proliferation of midbrain is encouraged. (Center) Immunoblots previously published in Huang *et al*. ^33^ (2017) showing a dose-dependent activation of the MAPK pathway (ERK1/2) by FGF8a, FGF8b, and FGF8b ^F32A^ in the BaF3 cell line. By introducing the F32A mutation or reducing the FGF8b concentration, the MAPK activation appears similar to FGF8a. Data are representative of three independent experiments. (Right) FGF8b dimerizes its receptor strongly, thus producing a robust and persistent signal that strongly induces En2, Spry1, and Gbx2 expression, which suppresses Otx2 expression. Under these settings, midbrain differentiates to cerebellum. Thus, quantitative differences in the stability of FGF-induced FGFR dimers translate into differences in the magnitude/duration of the intracellular signal, which in turn modify the transcriptional landscape and ultimately define the developmental response. Stronger receptor dimerization strength and higher signaling intensity are indicated by darker coloring. FGF, fibroblast growth factor; FGFR, fibroblast growth factor receptor; MAPK, mitogen-activated protein kinase.

The receptor-binding affinity of FGF is a key determinant of the strength and stability/durability of cell surface FGF-FGFR dimerization. Therefore, we infer that differences in neuronal activities of FGF8 isoforms stem from their different abilities to promote FGFR dimerization ^33^. Specifically, because FGF8b binds more tightly to its cognate FGFRs than FGF8a, FGF8b should induce the formation of FGF-FGFR dimers that are thermodynamically more stable and accordingly transmit a stronger and more sustained intracellular signal compared with FGF8a. It follows that, in the case of FGF8b, the magnitude of the MAPK signal should reach a threshold necessary for robust induction of *En2*, *Spry2*, and *Gbx2*, and the last of these should turn off *Otx2* expression ( Figure 3). According to our model, FGF-FGFR dimer stability plays a decisive role in the regulation of FGF signaling specificity by fine-tuning signaling intensity and duration. This in turn exerts a qualitative distinction by altering the landscape of activated downstream substrates/transcription factors. Taking into account the structural/biochemical data on FGF8a and FGF8b, we infer that the strength of FGF-induced FGFR dimerization varies to produce a signal with a magnitude that traverses a threshold for activating some downstream transcription factors while repressing others.

Our threshold model provides a molecular explanation for the distinct patterning potentials of FGF8a and FGF8b and also helps to explain published data linking ligand concentration and stability with biological outcome. In accordance with Le Chatelier’s principle, ligand concentration will directly determine the population/concentration of ligand-induced receptor dimers and hence the quality/quantity of the resulting intracellular signal. This explains why Sato *et al*. ^35^ (2001) were able to functionally convert FGF8b to FGF8a by simply reducing the expression level of FGF8b.

The threshold model can also account for why some RTKs are seemingly capable of employing signaling pathways in a cell type-dependent fashion. For example, in Swiss 3T3 cells, PDGFR redundantly uses the PI3K and PLCγ/PKC pathways to induce MAPK activation, whereas in Chinese hamster ovary (CHO) cells PDGFR strictly depends on PI3K to do so ^70^. This apparent CHO cell-dependent requirement of PI3K for PDGFR signaling can be reconciled with our threshold model. Specifically, compared with CHO cells, Swiss 3T3 cells express significantly higher levels of PDGFR such that, given sufficient ligand, they will display a much larger population of ligand-induced PDGFR dimers at the cell surface. The abundance of PDGFR dimers will maximally activate both the PI3K and the PLCγ/PKC pathways so much so that either pathway alone can generate sufficient signal to activate MAPK. As a result, elimination of the PI3K pathway would have little-to-no effect because the fully engaged PLCγ/PKC pathway alone can lead to sufficient activation of MAPK. By contrast, the signal flow from the PDGFR dimers in CHO cells is weak, such that only the additive effects of both the PI3K and the PLCγ/PKC pathways are capable of generating a strong enough signal to activate MAPK. Indeed, in the presence of lower physiological levels of PDGF, thus reducing the signal flow, PDGFR dimers require PI3K for activating MAPK in Swiss 3T3 cells ^70^.

The threshold model also explains the manner by which ligand stability impacts cell fate. Protein stability effectively determines the concentration of the functionally active pool of ligands capable of receptor binding and inducing dimerization. It follows that ligand stability also plays an important role in regulating cell specificity or diversity or both. Indeed, the instability of FGF1 vis-à-vis FGF2 underlies the former’s failure to induce sustained MAPK activation and expression of *NANOG*, a transcription factor necessary for the maintenance of pluripotency in stem cells ^71^.

**Figure 4.**
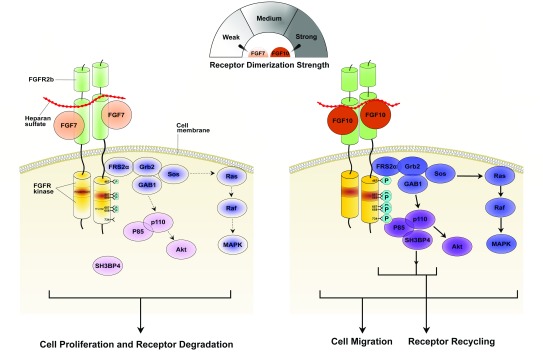
The “threshold model” can account for the activation of distinct pathways by FGF7 and FGF10 binding to FGFR2b. (Left) Relative to FGF10, FGF7 exhibits poor affinities for both FGFR2b and the heparan sulfate (HS) co-receptor and hence forms a weaker dimer with FGFR2b. Consequently, FGF7-FGFR2b dimers are less efficient in promoting transphosphorylation of A-loop tyrosines (necessary for kinase activation) and altogether fail to phosphorylate Y734 (necessary for the recruitment of distinct substrates and receptor recycling). Weaker kinase activation corresponds with a transient and overall weaker activation of MAPK that is further exacerbated by the inability of FGFR2b to recycle to the cell surface. (Right) FGF10 dimerizes its receptor strongly, promoting robust phosphorylation of A-loop tyrosines so as to transduce a prolonged signal. Y734 also becomes phosphorylated, thus enabling the recruitment of the P85-SH3BP4 complex in order to elicit receptor recycling and cell migration. Stronger receptor dimerization strength and higher signaling intensity and phosphorylation levels are indicated by darker coloring. A-loop, activation loop; FGF, fibroblast growth factor; FGFR, fibroblast growth factor receptor; MAPK, mitogen-activated protein kinase.

Furthermore, the model provides a molecular explanation for the functional dichotomy between FGF7 and FGF10 ligands, which signal through a shared receptor, FGFR2b. FGF10-FGFR2b binding generates a sustained MAPK signal and promotes cell migration, whereas FGF7-FGFR2b binding transmits a transient MAPK signal that leads to proliferation ^58^. Differences in intracellular signaling amplitude/duration and associated cellular behavior between these two ligands have been attributed to whether or not the ligands can induce phosphorylation of Y734 within the tyrosine kinase domain of FGFR2b. Specifically, unlike FGF10, FGF7 fails to induce phosphorylation of this site. Once phosphorylated, pY734 recruits a complex consisting of P85-PI3K and SH3BP4 to the receptor. This recruitment dictates recycling of the receptor to the plasma membrane, where the receptor continues to signal. Because FGF7 is unable to recruit this complex to FGFR2b, the receptor is destined for lysosomal degradation, thus allowing only a transient signal to propagate from the plasma membrane. We attribute the differential abilities of these two ligands to induce phosphorylation on tyrosine 734 to disparities in the stability of the FGFR2b dimers they induce. Indeed, relative to FGF7, FGF10 not only binds more tightly to FGFR2b but also has a higher affinity for the co-receptor heparan sulfate (HS) ^72^. As a result, FGF10-FGFR2b dimers are significantly more robust than FGF7-FGFR2b dimers, and FGF10-FGFR2b dimers enable more efficient transphosphorylation on the A-loop and on tyrosines involved in substrate recruitment, including Y734 ( Figure 4). It follows that differences in ligand-induced dimer stability result in both differences in the dynamics of intracellular signaling via A-loop tyrosine phosphorylation and in the choice of intracellular pathways. The latter phenomenon involves the phosphorylation of tyrosines that mediate the recruitment/activation of specific intracellular substrates.

In addition to transient and sustained modes of RTK signaling, recent studies have suggested pulsatile or oscillatory signaling as a third mode of the RTK response ^73– 77^. This can occur when a bistable system—that is, a system in which positive feedback amplifies downstream signals such that two stable equilibria are produced—is joined by negative feedback. The ensuing response is a system that periodically transitions between “off” and “on” states, achieving specificity via oscillating signals. Through the lens of the threshold model, we speculate that ligand-induced receptor dimer stability may determine the threshold necessary for reaching the next steady state, thereby indirectly determining the oscillatory response and cell fate. Indeed, in a bistable embryonic stem cell model, FGF-induced MAPK signaling levels have been shown to define the threshold value required for transitioning the system from a predominately epiblast (Epi)-like fate to primitive endoderm (PrE)-like cell differentiation ^78^. As these signaling levels are directly impacted by the receptor dimerization strength, it will be important to define what kinds of correlation exist between the on-/off-rates of dimerization for RTKs and the frequency or amplitude (or both) of the oscillating signals.

## Biased ligands lend support to the threshold model

For the threshold model to be robust, it should be possible to manipulate receptor dimerization strength so as to bias the expression of particular downstream factors, thereby achieving a desired cellular response. One way to do this would be to engineer mutated ligands that calibrate receptor dimerization thermodynamics. Indeed, we have recently shown that the mitogenic and metabolic activities of FGF1 can be uncoupled by introducing mutations into the HS binding site of FGF1 that dampen HS-assisted FGF1-FGFR dimerization. Although the mutant FGF1 lost its ability to induce proliferation of cultured cells *in vitro* and in mouse liver, it retained the full capacity of wild-type FGF1 to lower glucose levels *in vitro* and *in vivo* ^33^. Thus, a weak FGF1-FGFR dimer is sufficient to elicit a metabolic response, whereas a mitogenic response requires strong and sustained dimerization. SILAC (Stable Isotope Labeling by Amino acids in Cell culture)-based phosphoproteomic analysis of 3T3L1 fibroblasts (a pre-adipocyte cell line) that were stimulated with either wild-type or mutant FGF1 showed that the FGF1 mutant exhibited diminished FGFR phosphorylation on A-loop tyrosines and hence reduced total downstream signal flow compared with the wild type. Thus, quantitative differences in FGF1-FGFR dimerization strength translate into corresponding differences in intracellular signaling intensity and duration that determine mitogenic versus metabolic responses. We infer that there exists a certain threshold for FGF1-FGFR dimer stability such that, when crossed, the resulting signaling amplitude/duration enables the activation of intracellular pathways/transcription factors that are essential for a mitogenic signal but dispensable for a metabolic response ( Figure 2). The threshold model appears to be applicable to other RTK systems as well. For example, an engineered SCF variant with a reduced ability to dimerize its receptor, c-Kit, has been shown to have a decreased signaling amplitude that biases the activation of hematopoietic stem and progenitor cells (HSPCs) over mast cells ^32^.

A directive role for the physicodynamics of ligand–receptor dimerization in dictating cellular response is also implied by studies done on the cytokine receptor system. Members of this subfamily lack intrinsic kinase activity and therefore must rely on non-covalently associated JAKs to transmit their signals. For example, characterization of a cytokine erythropoietin (EPO) mutation known to cause severe anemia in humans showed that this mutation causes a selective impairment of downstream signal transduction in erythroid cells. Specifically, the mutation diminished JAK2-mediated phosphorylation of select downstream targets, including STAT1 and STAT3, while it had no impact on other targets such as STAT5 ^79^. With total internal reflection fluorescence (TIRF) microscopy of labeled receptor molecules on the cell surface, the EPO mutant was shown to incur an average 20% reduction in EPO receptor (EPOR) dimerization compared with the wild type, implying that EPO-EPOR dimerization strength modulates the quality of ensuing downstream signaling. In another study, mutants of the cytokine interleukin-2 (IL-2) were engineered to calibrate their signaling amplitudes by manipulating their affinities toward the β and γc subunits of the IL-2 receptor ^80^. These IL-2 mutants had an enhanced affinity for the β subunit of the receptor (IL-2Rβ) but a weakened affinity for the γc subunit, thereby impairing the dimerization dynamics of the receptor. The nature of the mutation at the γc interface is such that it is negatively correlated with IL-2 signaling strength and STAT5 phosphorylation. Thus, as heterodimerization is reduced, the signaling amplitude is reduced as well, and the most deleterious mutation culminates in a significant reduction of STAT5 phosphorylation and immunosuppression. Naturally, besides dimer stability, other factors—including the overall topology of the dimer—impact the signaling magnitude and duration. Indeed, by reorienting the geometry of receptor dimerization via the use of synthetic EPO surrogates called diabodies, it is possible to manipulate the signaling magnitude and choice of pathway ^81^. These diabodies impose large topological changes in the EPOR dimer that in turn affect pathway selection and the magnitude of the signal (STAT1 and STAT3 activation was decreased with the use of diabodies, while STAT5 activation remained unaffected). Thus, the threshold model appears to be pertinent to non-RTK systems as well.

The data mentioned above identify a positive correlation between the strength/stability of RTK dimerization and the duration/amplitude of the intracellular signal. On the other hand, studies on the EGF ligand receptor system have unexpectedly shown a negative correlation. For example, amphiregulin (AREG), a partial agonist of EGFR dimerization, generates higher levels of receptor phosphorylation and cell proliferation than the full agonist EGF ^82^. More recently, small-angle X-ray scattering (SAXS) showed that epiregulin (EREG)- and epigen (EPGN)-induced EGFR dimers are more than 60-fold weaker than those induced by transforming growth factor alpha (TGFα) and EGF in solution. Paradoxically, in cell-based studies, EREG and EPGN induced a more sustained activation of intracellular pathways compared with EGF or TGFα ^83^. Nonetheless, as expected, the sustained signal propagated by EREG- or EPGN-induced weak dimers leads to breast cancer cell differentiation, whereas the transient signal produced by EGF- or TGFα-induced strong dimers causes these cells to proliferate. To explain this disparity with respect to other RTK systems, the authors proposed that EREG- and EPGN-induced weak dimers may be unable to transphosphorylate a particular tyrosine residue on the receptor. This phosphorylated tyrosine is necessary for the recruitment of intracellular complexes that mediate receptor endocytosis/degradation and, consequently, signal termination. As a result, EREG- and EPGN-induced weak dimers may dwell longer on the cell surface and therefore transduce a more persistent signal than the EGF- and TGFα-induced stronger dimers. Indeed, it has been shown that each of the seven cognate ligands for EGFR signals for protein recruitment to the receptor in a distinctive way, and recruitment is dependent on ligand dosage ^84^. The justification for the counterintuitive negative correlation between receptor dimerization strength and the duration/amplitude of the intracellular signal may lie in the fact that, unlike all other RTKs, the EGFR system is activated via the formation of an asymmetric kinase dimer independent of A-loop tyrosine phosphorylation.

## Conclusions and future directions

Given the ubiquitous role of RTKs, understanding the mechanism by which they exert their distinct cellular functions is important for both advancing our comprehension of cellular biology and enabling strategies for treating human disease. Various models have been proposed over the years, and the most recent suggests that RTKs depend on receptor dimerization strength for determining cell decisions. Further use of engineered ligands should lead to a better delineation of the relationship between receptor dimerization and cellular response. Additionally, future studies should continue to explore more innovative ways to examine the receptor–ligand complex and cell surface receptors in general by engineering receptor mutants with varying levels of dimerization strength and topology. Moreover, future structural/biophysical work on the EGFR subfamily may reveal unknown conformational or kinetic differences within EGFR dimers that can account for their atypical mode of signaling compared to other RTK subfamilies.

Until very recently, quantification of RTK signaling could be done only *in vitro*. However, with the recent advent of a high-throughput phosphoproteomics platform, it is now possible to rapidly quantify the flow of RTK intracellular signaling *in vivo* ^85^. Likewise, novel fluorescent reporters and biomarkers, including genetically encoded FRET (Förster Resonance Energy Transfer) biosensors, are emerging as particularly effective tools for monitoring the activity of a given RTK in a non-invasive, *in vivo* fashion with enhanced spatiotemporal resolution ^86, 87^. Furthermore, advances in RNA sequencing and optimized computational methods are opening the door for genome-wide analyses, making it possible to determine the distinct cellular transcriptomes from each ligand–receptor signaling complex ^88^. Quantitative analyses of cell signaling mechanisms have been greatly enhanced by studies using *Drosophila*, making this an ideal system for pursuing additional genetic data on RTK signaling events ^89^. By applying these methods to study RTK signaling, one can reasonably hope for a better understanding of cell signaling in general as well as greater predictive power with respect to cellular decision-making. Ultimately, these efforts should lead to the development of novel and highly selective therapeutics that can be tailored for application to specific clinical conditions.

## References

[1] Böttcher RT Niehrs C: Fibroblast growth factor signaling during early vertebrate development. *Endocr Rev.* 2005;26(1):63–77. 10.1210/er.2003-0040 15689573

[2] Fu L John LM Adams SH: Fibroblast growth factor 19 increases metabolic rate and reverses dietary and leptin-deficient diabetes. *Endocrinology.* 2004;145(6):2594–603. 10.1210/en.2003-1671 14976145

[3] Carrera Silva EA Chan PY Joannas L: T cell-derived protein S engages TAM receptor signaling in dendritic cells to control the magnitude of the immune response. *Immunity.* 2013;39(1):160–70. 10.1016/j.immuni.2013.06.010 23850380 PMC4017237

[4] Jonker JW Suh JM Atkins AR: A PPARγ-FGF1 axis is required for adaptive adipose remodelling and metabolic homeostasis. *Nature.* 2012;485(7398):391–4. 10.1038/nature10998 22522926 PMC3358516

[5] Itoh N Ornitz DM: Fibroblast growth factors: from molecular evolution to roles in development, metabolism and disease. *J Biochem.* 2011;149(2):121–30. 10.1093/jb/mvq121 20940169 PMC3106964

[6] Turner N Grose R: Fibroblast growth factor signalling: from development to cancer. *Nat Rev Cancer.* 2010;10(2):116–29. 10.1038/nrc2780 20094046

[7] Lemmon MA Schlessinger J: Cell signaling by receptor tyrosine kinases. *Cell.* 2010;141(7):1117–34. 10.1016/j.cell.2010.06.011 20602996 PMC2914105

[8] De Meyts P Whittaker J: Structural biology of insulin and IGF1 receptors: implications for drug design. *Nat Rev Drug Discov.* 2002;1(10):769–83. 10.1038/nrd917 12360255

[9] Hubbard SR Till JH: Protein tyrosine kinase structure and function. *Annu Rev Biochem.* 2000;69:373–98. 10.1146/annurev.biochem.69.1.373 10966463

[10] Scapin G Dandey VP Zhang Z: Structure of the insulin receptor-insulin complex by single-particle cryo-EM analysis. *Nature.* 2018;556(7699):122–5. 10.1038/nature26153 29512653 PMC5886813

[11] Wardega P Heldin CH Lennartsson J: Mutation of tyrosine residue 857 in the PDGF beta-receptor affects cell proliferation but not migration. *Cell Signal.* 2010;22(9):1363–8. 10.1016/j.cellsig.2010.05.004 20494825

[12] DiNitto JP Deshmukh GD Zhang Y: Function of activation loop tyrosine phosphorylation in the mechanism of c-Kit auto-activation and its implication in sunitinib resistance. *J Biochem.* 2010;147(4):601–9. 10.1093/jb/mvq015 20147452

[13] Kazi JU Chougule RA Li T: Tyrosine 842 in the activation loop is required for full transformation by the oncogenic mutant FLT3-ITD. *Cell Mol Life Sci.* 2017;74(14):2679–88. 10.1007/s00018-017-2494-0 28271164 PMC5487891

[14] Endres NF Barros T Cantor AJ: Emerging concepts in the regulation of the EGF receptor and other receptor tyrosine kinases. *Trends Biochem Sci.* 2014;39(10):437–46. 10.1016/j.tibs.2014.08.001 25242369

[15] Hunter T: Signaling--2000 and beyond. *Cell.* 2000;100(1):113–27. 10.1016/S0092-8674(00)81688-8 10647936

[16] Schlessinger J Lemmon MA: SH2 and PTB domains in tyrosine kinase signaling. *Sci STKE.* 2003;2003(191):RE12. 10.1126/stke.2003.191.re12 12865499

[17] Schlessinger J: SH2/SH3 signaling proteins. *Curr Opin Genet Dev.* 1994;4(1):25–30. 10.1016/0959-437X(94)90087-6 8193536

[18] Schlessinger J: Cell signaling by receptor tyrosine kinases. *Cell.* 2000;103(2):211–25. 10.1016/S0092-8674(00)00114-8 11057895

[19] Simon MA Bowtell DD Dodson GS: Ras1 and a putative guanine nucleotide exchange factor perform crucial steps in signaling by the sevenless protein tyrosine kinase. *Cell.* 1991;67(4):701–16. 10.1016/0092-8674(91)90065-7 1934068

[20] Li N Batzer A Daly R: Guanine-nucleotide-releasing factor hSos1 binds to Grb2 and links receptor tyrosine kinases to Ras signalling. *Nature.* 1993;363(6424):85–8. 10.1038/363085a0 8479541

[21] Araki T Nawa H Neel BG: Tyrosyl phosphorylation of Shp2 is required for normal ERK activation in response to some, but not all, growth factors. *J Biol Chem.* 2003;278(43):41677–84. 10.1074/jbc.M306461200 12923167

[22] Marshall CJ: Specificity of receptor tyrosine kinase signaling: transient versus sustained extracellular signal-regulated kinase activation. *Cell.* 1995;80(2):179–85. 10.1016/0092-8674(95)90401-8 7834738

[23] Davis RJ: Signal transduction by the JNK group of MAP kinases. *Cell.* 2000;103(2):239–52. 10.1016/S0092-8674(00)00116-1 11057897

[24] Rawlings JS Rosler KM Harrison DA: The JAK/STAT signaling pathway. *J Cell Sci.* 2004;117(Pt 8):1281–3. 10.1242/jcs.00963 15020666

[25] Yang S Sharrocks AD Whitmarsh AJ: Transcriptional regulation by the MAP kinase signaling cascades. *Gene.* 2003;320:3–21. 10.1016/S0378-1119(03)00816-3 14597384

[26] Du K Montminy M: CREB is a regulatory target for the protein kinase Akt/PKB. *J Biol Chem.* 1998;273(49):32377–9. 10.1074/jbc.273.49.32377 9829964

[27] Campbell RA Bhat-Nakshatri P Patel NM: Phosphatidylinositol 3-kinase/AKT-mediated activation of estrogen receptor alpha: a new model for anti-estrogen resistance. *J Biol Chem.* 2001;276(13):9817–24. 10.1074/jbc.M010840200 11139588

[28] Menghini R Marchetti V Cardellini M: Phosphorylation of GATA2 by Akt increases adipose tissue differentiation and reduces adipose tissue-related inflammation: a novel pathway linking obesity to atherosclerosis. *Circulation.* 2005;111(15):1946–53. 10.1161/01.CIR.0000161814.02942.B2 15837948

[29] Vasudevan HN Soriano P: A Thousand and One Receptor Tyrosine Kinases: Wherein the Specificity? *Curr Top Dev Biol.* 2016;117:393–404. 10.1016/bs.ctdb.2015.10.016 26969991 PMC4789772

[30] Kratchmarova I Blagoev B Haack-Sorensen M: Mechanism of divergent growth factor effects in mesenchymal stem cell differentiation. *Science.* 2005;308(5727):1472–7. 10.1126/science.1107627 15933201

[31] Vasudevan HN Mazot P He F: Receptor tyrosine kinases modulate distinct transcriptional programs by differential usage of intracellular pathways. *eLife.* 2015;4:e07186. 10.7554/eLife.07186 25951516 PMC4450512

[32] Ho CCM Chhabra A Starkl P: Decoupling the Functional Pleiotropy of Stem Cell Factor by Tuning c-Kit Signaling. *Cell.* 2017;168(6):1041–1052.e18. 10.1016/j.cell.2017.02.011 28283060 PMC5526607

[33] Huang Z Tan Y Gu J: Uncoupling the Mitogenic and Metabolic Functions of FGF1 by Tuning FGF1-FGF Receptor Dimer Stability. *Cell Rep.* 2017;20(7):1717–28. 10.1016/j.celrep.2017.06.063 28813681 PMC5821125

[34] Sato T Nakamura H: The Fgf8 signal causes cerebellar differentiation by activating the Ras-ERK signaling pathway. *Development.* 2004;131(17):4275–85. 10.1242/dev.01281 15294862

[35] Sato T Araki I Nakamura H: Inductive signal and tissue responsiveness defining the tectum and the cerebellum. *Development.* 2001;128(13):2461–9. 11493563 10.1242/dev.128.13.2461

[36] Sato T Joyner AL Nakamura H: How does Fgf signaling from the isthmic organizer induce midbrain and cerebellum development? *Dev Growth Differ.* 2004;46(6):487–94. 10.1111/j.1440-169x.2004.00769.x 15610138

[37] Wright TJ Mansour SL: Fgf3 and Fgf10 are required for mouse otic placode induction. *Development.* 2003;130(15):3379–90. 10.1242/dev.00555 12810586

[38] Mariani FV Ahn CP Martin GR: Genetic evidence that FGFs have an instructive role in limb proximal-distal patterning. *Nature.* 2008;453(7193):401–5. 10.1038/nature06876 18449196 PMC2631409

[39] Znosko WA Yu S Thomas K: Overlapping functions of Pea3 ETS transcription factors in FGF signaling during zebrafish development. *Dev Biol.* 2010;342(1):11–25. 10.1016/j.ydbio.2010.03.011 20346941 PMC2866755

[40] Guo B Sharrocks AD: Extracellular signal-regulated kinase mitogen-activated protein kinase signaling initiates a dynamic interplay between sumoylation and ubiquitination to regulate the activity of the transcriptional activator PEA3. *Mol Cell Biol.* 2009;29(11):3204–18. 10.1128/MCB.01128-08 19307308 PMC2682013

[41] De Miguel MP Cheng L Holland EC: Dissection of the c-Kit signaling pathway in mouse primordial germ cells by retroviral-mediated gene transfer. *Proc Natl Acad Sci U S A.* 2002;99(16):10458–63. 10.1073/pnas.122249399 12140361 PMC124938

[42] Kissel H Timokhina I Hardy MP: Point mutation in kit receptor tyrosine kinase reveals essential roles for kit signaling in spermatogenesis and oogenesis without affecting other kit responses. *EMBO J.* 2000;19(6):1312–26. 10.1093/emboj/19.6.1312 10716931 PMC305672

[43] Tallquist MD French WJ Soriano P: Additive effects of PDGF receptor beta signaling pathways in vascular smooth muscle cell development. *PLoS Biol.* 2003;1(2):E52. 10.1371/journal.pbio.0000052 14624252 PMC261889

[44] Sudheer S Liu J Marks M: Different Concentrations of FGF Ligands, FGF2 or FGF8 Determine Distinct States of WNT-Induced Presomitic Mesoderm. *Stem Cells.* 2016;34(7):1790–800. 10.1002/stem.2371 27038343

[45] Ulu F Kim SM Yokoyama T: Dose-dependent functions of fibroblast growth factor 9 regulate the fate of murine XY primordial germ cells. *Biol Reprod.* 2017;96(1):122–33. 10.1095/biolreprod.116.143941 28395336 PMC5803787

[46] Reichman-Fried M Dickson B Hafen E: Elucidation of the role of breathless, a Drosophila FGF receptor homolog, in tracheal cell migration. *Genes Dev.* 1994;8(4):428–39. 10.1101/gad.8.4.428 8125257

[47] Li Y Roberts J AkhavanAghdam Z: Mitogen-activated protein kinase (MAPK) dynamics determine cell fate in the yeast mating response. *J Biol Chem.* 2017;292(50):20354–61. 10.1074/jbc.AC117.000548 29123025 PMC5733576

[48] Mohammadi M Dionne CA Li W: Point mutation in FGF receptor eliminates phosphatidylinositol hydrolysis without affecting mitogenesis. *Nature.* 1992;358(6388):681–4. 10.1038/358681a0 1379698

[49] Spivak-Kroizman T Mohammadi M Hu P: Point mutation in the fibroblast growth factor receptor eliminates phosphatidylinositol hydrolysis without affecting neuronal differentiation of PC12 cells. *J Biol Chem.* 1994;269(20):14419–23. 7514169

[50] Sorokin A Mohammadi M Huang J: Internalization of fibroblast growth factor receptor is inhibited by a point mutation at tyrosine 766. *J Biol Chem.* 1994;269(25):17056–61. 7516330

[51] Ong SH Guy GR Hadari YR: FRS2 proteins recruit intracellular signaling pathways by binding to diverse targets on fibroblast growth factor and nerve growth factor receptors. *Mol Cell Biol.* 2000;20(3):979–89. 10.1128/MCB.20.3.979-989.2000 10629055 PMC85215

[52] Hadari YR Gotoh N Kouhara H: Critical role for the docking-protein FRS2 alpha in FGF receptor-mediated signal transduction pathways. *Proc Natl Acad Sci U S A.* 2001;98(15):8578–83. 10.1073/pnas.161259898 11447289 PMC37478

[53] Hadari YR Kouhara H Lax I: Binding of Shp2 tyrosine phosphatase to FRS2 is essential for fibroblast growth factor-induced PC12 cell differentiation. *Mol Cell Biol.* 1998;18(7):3966–73. 10.1128/MCB.18.7.3966 9632781 PMC108981

[54] Valius M Kazlauskas A: Phospholipase C-gamma 1 and phosphatidylinositol 3 kinase are the downstream mediators of the PDGF receptor's mitogenic signal. *Cell.* 1993;73(2):321–34. 10.1016/0092-8674(93)90232-F 7682895

[55] Bain J Plater L Elliott M: The selectivity of protein kinase inhibitors: a further update. *Biochem J.* 2007;408(3):297–315. 10.1042/BJ20070797 17850214 PMC2267365

[56] Royal I Fournier TM Park M: Differential requirement of Grb2 and PI3-kinase in HGF/SF-induced cell motility and tubulogenesis. *J Cell Physiol.* 1997;173(2):196–201. 10.1002/(SICI)1097-4652(199711)173:2<196::AID-JCP20>3.0.CO;2-D 9365521

[57] Belleudi F Leone L Nobili V: Keratinocyte growth factor receptor ligands target the receptor to different intracellular pathways. *Traffic.* 2007;8(12):1854–72. 10.1111/j.1600-0854.2007.00651.x 17944804

[58] Francavilla C Rigbolt KT Emdal KB: Functional proteomics defines the molecular switch underlying FGF receptor trafficking and cellular outputs. *Mol Cell.* 2013;51(6):707–22. 10.1016/j.molcel.2013.08.002 24011590

[59] Brewer JR Mazot P Soriano P: Genetic insights into the mechanisms of Fgf signaling. *Genes Dev.* 2016;30(7):751–71. 10.1101/gad.277137.115 27036966 PMC4826393

[60] Grabner A Amaral AP Schramm K: Activation of Cardiac Fibroblast Growth Factor Receptor 4 Causes Left Ventricular Hypertrophy. *Cell Metab.* 2015;22(6):1020–32. 10.1016/j.cmet.2015.09.002 26437603 PMC4670583

[61] Yu C Wang F Jin C: Independent repression of bile acid synthesis and activation of c-Jun N-terminal kinase (JNK) by activated hepatocyte fibroblast growth factor receptor 4 (FGFR4) and bile acids. *J Biol Chem.* 2005;280(18):17707–14. 10.1074/jbc.M411771200 15750181

[62] Cirit M Haugh JM: Data-driven modelling of receptor tyrosine kinase signalling networks quantifies receptor-specific potencies of PI3K- and Ras-dependent ERK activation. *Biochem J.* 2012;441(1):77–85. 10.1042/BJ20110833 21943356 PMC3687362

[63] Shaoul E Reich-Slotky R Berman B: Fibroblast growth factor receptors display both common and distinct signaling pathways. *Oncogene.* 1995;10(8):1553–61. 7731710

[64] Furdui CM Lew ED Schlessinger J: Autophosphorylation of FGFR1 kinase is mediated by a sequential and precisely ordered reaction. *Mol Cell.* 2006;21(5):711–7. 10.1016/j.molcel.2006.01.022 16507368

[65] Chen H Xu CF Ma J: A crystallographic snapshot of tyrosine *trans*-phosphorylation in action. *Proc Natl Acad Sci U S A.* 2008;105(50):19660–5. 10.1073/pnas.0807752105 19060208 PMC2605006

[66] Lew ED Furdui CM Anderson KS: The precise sequence of FGF receptor autophosphorylation is kinetically driven and is disrupted by oncogenic mutations. *Sci Signal.* 2009;2(58):ra6. 10.1126/scisignal.2000021 19224897 PMC2755185

[67] Huang Z Chen H Blais S: Structural mimicry of a-loop tyrosine phosphorylation by a pathogenic FGF receptor 3 mutation. *Structure.* 2013;21(10):1889–96. 10.1016/j.str.2013.07.017 23972473 PMC3839590

[68] Olsen SK Li JY Bromleigh C: Structural basis by which alternative splicing modulates the organizer activity of FGF8 in the brain. *Genes Dev.* 2006;20(2):185–98. 10.1101/gad.1365406 16384934 PMC1356110

[69] Katahira T Sato T Sugiyama S: Interaction between *Otx2* and *Gbx2* defines the organizing center for the optic tectum. *Mech Dev.* 2000;91(1–2):43–52. 10.1016/S0925-4773(99)00262-2 10704829

[70] Duckworth BC Cantley LC: Conditional inhibition of the mitogen-activated protein kinase cascade by wortmannin. Dependence on signal strength. *J Biol Chem.* 1997;272(44):27665–70. 10.1074/jbc.272.44.27665 9346906

[71] Chen G Gulbranson DR Yu P: Thermal stability of fibroblast growth factor protein is a determinant factor in regulating self-renewal, differentiation, and reprogramming in human pluripotent stem cells. *Stem Cells.* 2012;30(4):623–30. 10.1002/stem.1021 22213113 PMC3538808

[72] Makarenkova HP Hoffman MP Beenken A: Differential interactions of FGFs with heparan sulfate control gradient formation and branching morphogenesis. *Sci Signal.* 2009;2(88):ra55. 10.1126/scisignal.2000304 19755711 PMC2884999

[73] Volinsky N Kholodenko BN: Complexity of receptor tyrosine kinase signal processing. *Cold Spring Harb Perspect Biol.* 2013;5(8):a009043. 10.1101/cshperspect.a009043 23906711 PMC3721286

[74] Nakayama K Satoh T Igari A: FGF induces oscillations of Hes1 expression and Ras/ERK activation. *Curr Biol.* 2008;18(8):R332–4. 10.1016/j.cub.2008.03.013 18430630

[75] Shankaran H Ippolito DL Chrisler WB: Rapid and sustained nuclear-cytoplasmic ERK oscillations induced by epidermal growth factor. *Mol Syst Biol.* 2009;5:332. 10.1038/msb.2009.90 19953086 PMC2824491

[76] Shin SY Rath O Choo SM: Positive- and negative-feedback regulations coordinate the dynamic behavior of the Ras-Raf-MEK-ERK signal transduction pathway. *J Cell Sci.* 2009;122(Pt 3):425–35. 10.1242/jcs.036319 19158341

[77] Hu H Goltsov A Bown JL: Feedforward and feedback regulation of the MAPK and PI3K oscillatory circuit in breast cancer. *Cell Signal.* 2013;25(1):26–32. 10.1016/j.cellsig.2012.09.014 23000339

[78] Schröter C Rué P Mackenzie JP: FGF/MAPK signaling sets the switching threshold of a bistable circuit controlling cell fate decisions in embryonic stem cells. *Development.* 2015;142(24):4205–16. 10.1242/dev.127530 26511924 PMC4689219

[79] Kim AR Ulirsch JC Wilmes S: Functional Selectivity in Cytokine Signaling Revealed Through a Pathogenic *EPO* Mutation. *Cell.* 2017;168(6):1053–1064.e15. 10.1016/j.cell.2017.02.026 28283061 PMC5376096

[80] Mitra S Ring AM Amarnath S: Interleukin-2 activity can be fine tuned with engineered receptor signaling clamps. *Immunity.* 2015;42(5):826–38. 10.1016/j.immuni.2015.04.018 25992859 PMC4560365

[81] Moraga I Wernig G Wilmes S: Tuning cytokine receptor signaling by re-orienting dimer geometry with surrogate ligands. *Cell.* 2015;160(6):1196–208. 10.1016/j.cell.2015.02.011 25728669 PMC4766813

[82] Macdonald-Obermann JL Pike LJ: Different epidermal growth factor (EGF) receptor ligands show distinct kinetics and biased or partial agonism for homodimer and heterodimer formation. *J Biol Chem.* 2014;289(38):26178–88. 10.1074/jbc.M114.586826 25086039 PMC4176247

[83] Freed DM Bessman NJ Kiyatkin A: EGFR Ligands Differentially Stabilize Receptor Dimers to Specify Signaling Kinetics. *Cell.* 2017;171(3):683–695.e18. 10.1016/j.cell.2017.09.017 28988771 PMC5650921

[84] Ronan T Macdonald-Obermann JL Huelsmann L: Different Epidermal Growth Factor Receptor (EGFR) Agonists Produce Unique Signatures for the Recruitment of Downstream Signaling Proteins. *J Biol Chem.* 2016;291(11):5528–40. 10.1074/jbc.M115.710087 26786109 PMC4786695

[85] Humphrey SJ Azimifar SB Mann M: High-throughput phosphoproteomics reveals *in vivo* insulin signaling dynamics. *Nat Biotechnol.* 2015;33(9):990–5. 10.1038/nbt.3327 26280412

[86] González-Vera JA Morris MC: Fluorescent Reporters and Biosensors for Probing the Dynamic Behavior of Protein Kinases. *Proteomes.* 2015;3(4):369–410. 10.3390/proteomes3040369 28248276 PMC5217393

[87] Zhang Q Huang H Zhang L: Visualizing Dynamics of Cell Signaling *In Vivo* with a Phase Separation-Based Kinase Reporter. *Mol Cell.* 2018;69(2):334–346.e4. 10.1016/j.molcel.2017.12.008 29307513 PMC5788022

[88] Spies D Ciaudo C: Dynamics in Transcriptomics: Advancements in RNA-seq Time Course and Downstream Analysis. *Comput Struct Biotechnol J.* 2015;13:469–77. 10.1016/j.csbj.2015.08.004 26430493 PMC4564389

[89] Shilo BZ: New Twists in *Drosophila* Cell Signaling. *J Biol Chem.* 2016;291(15):7805–8. 10.1074/jbc.R115.711473 26907691 PMC4824987

